# C3 glomerulopathy is highly prevalent in French Polynesia

**DOI:** 10.1016/j.jtauto.2024.100254

**Published:** 2024-10-29

**Authors:** Nelly Candela, Nicolas Benichou, Mathilde Lefebvre, Lorraine Gueguen, Paula Vieira-Martins, Carine El Sissy, Hervé Sartelet, Pascale Testevuide, Ronan Delaval, Stanislas Faguer

**Affiliations:** a Service de Néphrologie, Centre Hospitalier du Taaone, Tahiti, French Polynesia; b Département de Néphrologie et Transplantation d'organes, Centre de Référence des maladies rénales rares, Centre Hospitalier Universitaire de Toulouse, Toulouse, France; c Service de Néphrologie, Groupe Hospitalier Privé Ambroise Paré Hartmann, Neuilly sur Seine, France; d Laboratoire d’Immunologie, Hôpital Européen Georges Pompidou, Assistance Publique des Hôpitaux de Paris, Paris, France; e Laboratoire d'anatomopathologie, CHU de Nancy, Nancy, France

**Keywords:** C3 glomerulonephritis, Acute post-infectious glomerulopathy, Complement alternative pathway, Complement factor I

## Abstract

**Objective:**

To compare the natural history of C3 glomerulopathy (C3G) to acute post-infectious glomerulonephritis (APIGN) in a cohort of patients with a relative homogeneity of environment conditions and genetic background.

**Methods:**

We retrospectively reviewed the characteristics of all patients with biopsy proven C3G or APIGN referred in 2013–2019 to the only renal unit in French Polynesia.

**Results:**

Point prevalence of C3G is ∼23 cases per 100,000 inhabitants. A recurrent variation of CFI (p.Arg406His) was identified at the heterozygous state in 4/8 (50 %) patients with C3G but its pathogenicity remain elusive. Characteristics at presentation and kidney outcomes were roughly similar between C3G (n = 16) and APIGN (n = 20), excepted for the presence of humps on kidney biopsy.

**Conclusions:**

C3G is highly prevalent in French Polynesia suggesting specific genetic or environmental susceptibility factors. Systematic diagnosis workflow should be proposed to all patients with C3 predominant glomerulonephritis.

## Abbreviations

**ACP**: alternative complement pathway
**APIGN**: acute post-infection glomerulonephritis
**C3G**: C3 glomerulopathy
**CFB**: complement factor B
**CFI**: complement factor I
**eGFR**: estimated glomerular filtration rate
**Ig-C3G**: monoclonal gammopathy-related C3 glomerulopathy
**M-spike**: monoclonal spike
**MGUS**: monoclonal gammopathy of unknown significance

## 1 Introduction

C3 glomerulopathy (C3G) and acute post-infection glomerulonephritis (APIGN) are two kidney diseases driven, among other mechanisms, by uncontrolled activation of complement pathways [1]. These two kinds of glomerulonephritis are characterized by massive C3 deposition in glomeruli associated or not with immunoglobulin deposits. In APIGN, C3 deposits are typically bright with sub-epithelial localization, whereas in C3G, deposits are semi-linear or granular and are mainly localized in the mesangium or within the basement membrane. In both conditions, on optical examination various patterns can be seen including endocapillary or mesangial proliferation, membranoproliferative aspect, or crescentic lesions [2].

In C3G, activation of the alternative complement pathway (ACP) relied on several acquired (e.g., C3Nef, anti-factor H or anti-C3 antibodies, and monoclonal immunoglobulin (mIg-C3)) or inherited (e.g.*,* mutations in *C3, CFB*, *CFH*, *CFI*) disorders [3]. In APIGN, the role of acquired anti-factor B antibodies was recently demonstrated in a cohort of children but these antibodies are also identified in patients with C3G or immune complexes-mediated membranous and proliferative glomerulonephritis (Ig-MPGN) [4,5]. In addition, in a subset of APIGN with unfavorable kidney outcome and low serum C3 beyond 3 months, inherited dysregulation of the alternative complement pathway was identified, suggesting a continuum between the two disorders or common molecular mechanisms [6]. Accordingly, C3G development or relapse frequently occurs after a bacterial or viral infection [3].

In this study, we aimed to characterize the presentation and outcome of C3G in patients from French Polynesia, a specific population highly exposed to infections and with relatively homogeneous genetic background. French Polynesia is composed of five archipelagos (118 islands). Its population is characterized by insularity, a homogeneous genetic background, and a high incidence of bacterial infections, especially cutaneous infections. We reviewed the phenotype and molecular determinants of biopsy proven C3G and APIGN in a cohort of 36 Polynesian patients and assessed the incidence of a specific CFI variation in a well-defined geographical region.

## 2 Patients and methods

### 2.1 Inclusion criteria

In this retrospective monocentric study, we included all individuals followed between January 2013 and March 2019 at the Taaone Hospital, the only nephrology unit in French Polynesia, who fulfilled the two following criteria of inclusion: (*i*) a native kidney biopsy available and (*ii*) the identification of C3 deposits in glomeruli with at least 2+ intensity. Patients with a diagnosis of systemic erythematosus lupus, cryoglobulinemia, IgA nephropathy, primary focal and segmental glomerulonephritis, Ig-MPGN or membranoproliferative glomerulonephritis with monoclonal Ig deposits were excluded. All biopsy reports were reviewed by investigators (NC, NB, and SF).

Patients fulfilling the inclusion criteria were divided into 2 groups according to the final diagnosis (C3G or APIGN). APIGN was retained in patients with clinical evidence of bacterial infection or positive microbiological screening. C3G was retained in patients without any evidence of recent or ongoing infection, and negative microbiological screening, and with isolated C3 staining or predominant staining (with a difference of at least 2+ of magnitude between the staining intensity of C3 and immunoglobulins) [7,8]. Final diagnosis was retained after in-depth review of the clinical charts by two investigators, who followed the patients (RD) or not (SF).

Data collection was conducted according to the declaration of Helsinki, as revised in 2004. Regarding the French law on retrospective studies relying on data, written informed consent was waived.

### 2.2 Kidney pathology

Renal biopsy included light microscopy (staining with hematoxylin and eosin, periodic acid-Schiff, Masson's trichrome and Jones methenamine silver) and immunofluorescence (3 μm cryostat sections stained with polyclonal antibodies to IgG, IgM, IgA, C3, C1q, kappa, lambda (rabbit, polyclonal, Agilent). Electron microscopy was available only in a subset of patients of C3G and no patient with APIGN.

### 2.3 Immunological assays

EDTA plasma samples were obtained from all tested patients. Plasma protein concentrations of C3, C4 were measured by nephelometry (Dade Behring, Deerfield, IL, USA). Soluble C5b-9 level determination was done using the MicroVue sC5b-9 Plus EIA Assay (Quidel, San Diego, CA), according to manufacturer's instructions. Presence of anti–factor H, anti-factor B and anti-C3b antibodies was detected by using an enzyme-linked immunosorbent assay (ELISA), as previously described elsewhere [9,10]. The C3NeF stabilization assay was performed using hemolytic assays as previously described elsewhere [11]. Direct sequencing or NGS of all *CFH*, *CFI*, *C3*, *CFB* exons was performed as previously described elsewhere [11]. Screening for complex genetic alterations affecting *CFH*, *CFHR1*, and *CFHR3* secondary to nonallelic homologous recombination was undertaken using multiplex ligation–dependent probe amplification from MRC Holland (www.mlpa.com) and homemade probes, as previously described elsewhere [12].

### 2.4 Statistical analyses

Continuous variables were shown as median and minimum/maximum. Binary variables were shown as number and percentage. Data from C3G and APIGN were compared using the Kruskal-Wallis test (continuous variables) or the Chi-square test (discontinuous variables). A p-value below 0.05 was considered significant.

To be compared to the point prevalence reported in other studies [3], the values was calculated as (n cases/n referral population) × (population average life expectancy – median or mean age of case patients)/n years of data collection. In 2020, life expectancy in French Polynesia was 74.7 years for men and 79.1 years for women (Institute for statistics of French Polynesia; https://www.ispf.pf/publication/1256). The number of inhabitants was 279,300.

## 3 Results

**Fig. 1:**
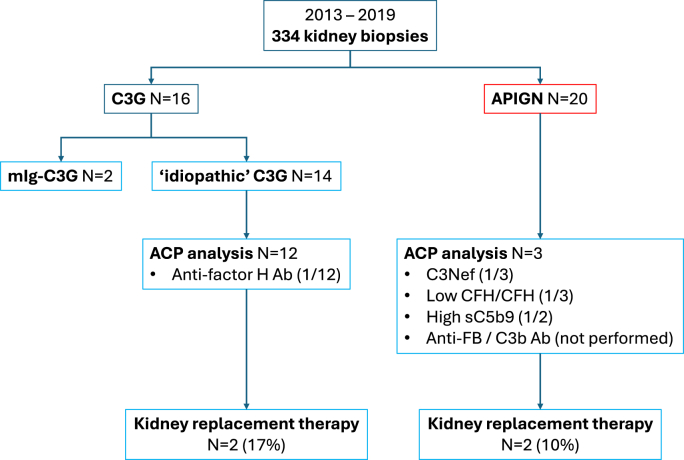
Flow chart of the study.

Between 2013 and 2019, a kidney biopsy was performed in 334 patients in French Polynesia. Among them, 36 patients fulfilled the inclusion criteria. A final diagnosis of C3G was retained in 16 patients (including monoclonal Ig-related C3G in two) and APIGN in 20 patients (Fig. 1). The point prevalence of C3G was thus estimated around 23 for 100,000 individuals.

### 3.1 Clinical characteristics

Among APIGN patients, active infection was proven in 14 patients (cutaneous infection n = 9, angina n = 2, urinary infection n = 2, syphilis n = 1). In the others, APIGN developed in the days or weeks following infections (dental infection or infections of unknown origin). It is of note that an M-spike was identified in 4 APIGN patients (MGUS in all).

mIg-C3G was diagnosed in 2 patients aged 75 and 77 years who had indolent multiple myeloma or monoclonal gammopathy of undetermined significance (MGUS), including one with low serum level of C3 (sC3, 0.66 g/L). One patient with mIg-C3G received plasma cell-directed chemotherapy (bortezomib plus dexamethasone) and reached partial remission. The second patient received nephroprotection only and had stable eGFR.

**Fig. 2:**
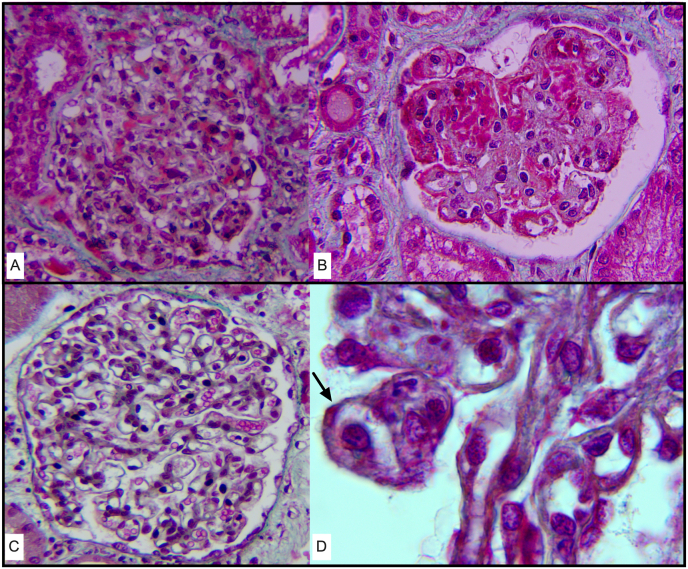
**Kidney biopsy (hematoxylin-eosin staining) of 4 patients with C3G or APIGN. A-B.** Diffuse thickening of the glomerular capillary walls, along with the deposition of C3 (C3G pattern) **C-D.** Typical features of humps (well-defined rounded deposits outside of the capillary walls; black arrow) (APIGN pattern).

Thereafter, we compared characteristics of the 14 ‘idiopathic’ C3G (i.e. after exclusion of mIg-C3G) and 20 APIGN patients. As shown in Table 1, few parameters discriminated C3G and APIGN at presentation. The severity of renal dysfunction was similar in C3G and APIGN patients, as well as the level of urinary protein to creatinine ratio and sC3. On kidney biopsy, the only parameters that discriminated patients were the presence of humps (8/20 vs. 0/14, p = 0.001), the identification of double contours of the glomerular basement membrane (4/20 vs. 8/14, p = 0.025), and the concomitant deposition of IgG (10/20 vs. 1/14, p = 0.02). Frequencies of extracapillary crescents and endocapillary proliferation were similar between conditions. Fig. 2 shows typical findings of C3G (sub-endothelial and mesangial deposits; 1-B) and APIGN (humps; C-D) on kidney biopsy.

**Table 1:** **Characteristics of patients with acute post-infectious glomerulonephritis (n** = **20) or C3 glomerulopathy (n** = **14).***APIGN*, acute post-infectious glomerulonephritis; *C3G*, C3 glomerulopathy; SD, standard deviation.

Characteristics	APIGN N = 20	C3G N = 14
Male (n, %)	17 (85)	10 (74)
Age (years, mean ± SD)	47 ± 23	49 ± 17
*Medical History*		
Hypertension (n, %)	11 (55)	6 (43)
Diabetes mellitus (n, %)	5 (25)	3 (21)
Known chronic kidney disease (n, %)	7 (35)	3 (21)
*Biological tests*		
Serum albumin (g/L, mean ± SD)	28 ± 4	29 ± 6
Serum creatinine (mg/L, mean ± SD)	28.9 ± 15	29.1 ± 29
Urinary protein/creatinine ratio (g/g; mean ± SD)	6.7 ± 5	6.2 ± 6
C3 (g/L, mean ± SD)	0.60 ± 0.42	0.63 ± 0.35
C4 (g/L, mean ± SD)	0.29 ± 0.11	0.29 ± 0.10
*Kidney biopsy* (n, %)		
Mesangial proliferation	13 (65)	11 (78)
Endocapillary proliferation	16 (80)	8 (57)
Crescentic proliferation	11 (55)	6 (43)
Humps	8 (40)	0
Double contours of GBM	4 (20)	8 (57)
Immune deposits		
IgG	10 (50)	1 (7)
IgA	3 (15)	1 (7)
IgM	2 (10)	0
C1q	3 (15)	1 (7)

### 3.2 Alternative complement pathway analyses

Functional analyses of the ACP were available in 15 patients (APIGN n = 3, C3G n = 12). Among the 3 APIGN patients with available data, a C3 nephritic factor was identified in one patient with syphilis. Serum levels of factor I and factor H were normal in 3/3 and reduced (48 % of normal value) in 1/3 patients, respectively. Soluble C5b9 was increased (946 ng/mL) in one out of the two APIGN patients with available data. Membrane expression of CD46 was normal in one tested patient. No patient had anti-factor H antibody. A search for anti-factor B antibodies was not performed at the time patients were followed.

Among C3G patients, an anti-factor H antibody was identified in one patient, whereas none had C3 nephritic factor or anti-factor I antibody. Search for anti-factor B and anti-C3b antibody was retrospectively performed in four patients (samples were collected at the time of kidney biopsy) and was negative in all. Membrane expression of CD46 was normal in the seven tested patients.

Molecular analysis of complement genes was performed in 10 patients (APIGN n = 2, C3G n = 8) and identified a recurrent variation in *CFI* (R406H). This variation was identified in 4/8 patients with C3G (50 %; heterozygous state) and in 0/2 with APIGN. This variation was also identified in one patient with mIg-C3G.

### 3.3 Treatments and kidney outcome

At presentation, renal replacement therapy was required in 2/16C3G patients (12.5 %) and 2/20 APIGN patients (10 %). Among APIGN patients, 8/20 (40 %) received glucocorticoids in addition to antibiotics, and one (5 %) also received mycophenolate mofetil (MMF) because of initial diagnosis uncertainty. Among C3G patients, 6/16 received glucocorticoids (37.5 %) and 3 received MMF (19 %). None received eculizumab. One patient with mIg-C3G received plasma cell-directed chemotherapy (bortezomib plus dexamethasone) and reached complete hematological response with stable kidney function (serum creatinine 22 mg/dL). In the second mIg-C3G patient, C3 levels normalized spontaneously.

After a mean follow-up of 8 months, eGFR were 84 ± 68 and 74 ± 54 mL/min/1.73 m^2^ in APIGN and C3G patients, respectively (p = 0.67). Kidney replacement therapy was required in 2 idiopathic C3G patients (17 %) and 2 APIGN patients (10 %).

## 4 Discussion

Characterizing the presentation and outcomes of kidney diseases in populations with homogeneous genetic background and environmental exposure may help to refine their natural histories or identify specific risk factors of progression. Recently, we demonstrated that standardized diagnosis workup, including immunological and genetic testing and kidney biopsy in indigenous people with kidney disease of unknown origin dramatically improves the rate of specific kidney diseases diagnosis, beyond diabetic or hypertensive nephropathies [13].

In this retrospective study, we extensively reviewed all the kidney biopsies performed in French Polynesia over a 6-year period and could estimate the point prevalence of C3G to ∼23 cases per 100,000 inhabitants, a picture twofold higher than what was previously reported in Europe and up to 40-fold compared to US prevalence [3], suggesting specific genetic or environmental risk factors. This prevalence is probably underestimated, given the lack of systematic kidney biopsy up to recently.

Rare variants in *CFI* are a known cause of C3G [14]. Factor I is a plasma glycoprotein regulator of the alternative pathway of the complement system. Factor I regulates complement activation by inactivating C3b and C4b through proteolytic cleavage in the presence of one of its cofactor proteins (Factor H, membrane cofactor protein (MCP, CD46), C4b-binding protein (C4BP) or complement receptor 1 (CR1)).

Here, we identified the R406H CFI variant in 50 % of C3G patients with genetic analyses. The allele frequency of the R406H variation is below 1 % in Europeans, Africans, Africans/Americans, and Latinos, but increased up to 13.2 % in East Asians (*GnomAD.broadinstitute.org/variant/4-110667590-C-T*) (Table 2).

**Table 2:** Characteristics of the p.Arg406His variant of *CFI*.

Complement factor I (*CFI; ENST00000394634.7*): c.1217G > A, p.Arg406His In silico prediction SIFT: tolerated, REVEL: pathogenic, FATHMM: pathogenic, LRT: neutral, PLI: tolerated Ex vivo analyses of the p.Arg406His variant *Kavannagh* et al. [15,16] Normal C3b cleavage by factor H or C4b cleavage by C4BP (normal activity of mutated factor I) *Java* et al. [17] Reduced rate of C3b cleavage by factor H and CR1 (decrease activity of mutated CFI) *Kavannagh* et al. [16], *De Jong* et al. [18] Normal factor I dosage despite mutation *Hallam* et al. [19] No functional effect on complement activation	

**Origin**	**Allele frequency (GnomAD®)**
African/African-American	0.1 %
Middle Eastern	0.6 %
Admixed American	0.4 %
European (non-Finnish)	0.01 %
European (Finnish)	2.3 %
East Asian	9.8 %
South Asian	4.1 %

Several studies have reported the functional evaluation of cofactor activity of the p.Arg406His *CFI* variant with contradictory results. Formerly, no reduce rate of C3b cleavage by factor H or C4b cleavage by C4BP were observed in functional analysis with this variant compared to wild type, thus the cofactor activity of this variant was maintained [15,16]. On the other hand, Java et al. have shown a reduced rate of C3b cleavage by factor H and CR1, and a maintained rate of C3b cleavage by MCP compared to wild-type CFI [17]. Both recombinant studies and CFI dosage in patients carrying the p.Arg406His variant have shown no decreased expression of CFI [16,18]. The p.Arg406His variant is classified as benign (ACMG, 2015 BP4) by several in silico predictors, but possibly damaging by Polyphen-2. Finally, Hallam et al. elegantly showed *in vitro* that the p.Arg406His variant has no functional effect on complement activation [19]. Also, the high incidence of the p.Arg406His variant in French Polynesia (>20 %) does not support the pathogenic role of this variant [20].

A second key finding of this study is the almost similar presentation of APIGN and C3G, the kidney biopsy being the only discriminative test. Given the high incidence of chronic skin wounds and infections in French Polynesia, owing to the high frequency of filariasis-related elephantiasis or to the high-risk lifestyle (similarly to other tropical areas), kidney biopsy and screening for ACP activation should be proposed to all patients with acute or subacute glomerulonephritis. Among the limitations, the lack of systematic screening for anti-factor B antibodies did not allow us to test the value of this non-invasive test to discriminate patients with APIGN or GC3. In addition, ACP analysis was not available in all patients, and not performed in first-degree relatives, probably under-estimating the rate of acquired or inherited abnormalities.

Third, kidney outcome was similar between APIGN and GC3 patients with ∼10 % of patients requiring chronic kidney replacement after one year of follow-up. Lack of compliance and loss of follow-up were frequent pitfalls. Optimal kidney protection with renin-angiotensin-aldosterone inhibitors (and now gliflozins) or immunosuppressive regimen (e.g., mycophenolate mofetil and glucocorticoids) [3] was not reached in all patients, blurring its evaluation in the long term.

## 5 Conclusions

We have shown that C3G is highly prevalent in French Polynesia and that a recurrent *CFI* variant may contribute to its development. The presentation and outcome of C3G and APIGN, two forms of complement-mediated glomerulopathy, largely overlap. Whether the p.Arg406His *CFI* variant influences the progression of kidney disease outside the setting of C3G remains to be explored.

## Funding information

The authors declare they received no specific funding for the present study.

## CRediT authorship contribution statement

**Nelly Candela:** Writing – review & editing, Investigation, Data curation. **Nicolas Benichou:** Writing – review & editing, Investigation. **Mathilde Lefebvre:** Writing – review & editing, Investigation, Data curation. **Lorraine Gueguen:** Writing – review & editing, Investigation. **Paula Vieira-Martins:** Writing – review & editing, Investigation. **Carine El Sissy:** Writing – review & editing, Investigation. **Hervé Sartelet:** Investigation, Writing – review & editing. **Pascale Testevuide:** Writing – review & editing, Investigation. **Ronan Delaval:** Writing – review & editing, Investigation, Conceptualization. **Stanislas Faguer:** Writing – original draft, Methodology, Investigation, Data curation, Conceptualization.

## Declaration of competing interest

The authors declare the following financial interests/personal relationships which may be considered as potential competing interests:SF received personal fees for consultancy from Abionyx Pharma, CSL-VIFOR and 10.13039/100004336Novartis SA, for scientific advisory boards from CSL-Vifor, Sanofi-Genzyme, Alexion-AstraZeneca, and for lecture from 10.13039/100004702Baxter and CSL-Vifor. Other authors declared no conflict of interest.

## Data Availability

Data will be made available on request.
